# Academic Community Medicine in 21^st^ Century: Challenges and Opportunities

**DOI:** 10.4103/0970-0218.45367

**Published:** 2009-01

**Authors:** Rajesh Kumar

**Affiliations:** PGIMER School of Public Health, Chandigarh 160 012, India. E-mail: dr.rajeshkumar@gmail.com

The last century witnessed the birth, growth, and maturation of community medicine as a scientific discipline. However, changes in the health scenario at the turn of the century posed several challenges to academic community medicine. Due to demographic and epidemiologic transition the disease burden of chronic non-communicable diseases and injuries is rising whereas communicable diseases, malnutrition, maternal and child health problems are yet to be overcome. With rapid industrial growth, environmental and occupational health problems are also likely to pose a bigger threat to population health. Moreover, the existing health system, which is based on a biomedical model of disease prevention and control, is not adequately geared to face these challenges.

The core function of academic community medicine is to study the health and disease in defined communities, identify their health needs, plan and evaluate programs so as to effectively meet their health needs. However, in the present era of globalization, social policies at the national and international level have a profound influence on the health of communities. This calls for new approaches to tackle determinants of health at the local and global level. To face the emerging challenges community physicians not only need thorough knowledge of epidemiological and bio-statistical methods, but also need to know the relevant aspects of the social, political, economic and environmental sciences, and the principles of administration and management. Applying this knowledge to identify health determinants, development of public policies and plans to address these determinants, management and evaluation of health programs requires a variety of skills, a challenging task indeed. This calls for integration of biomedical, ecological and sociological approaches with academic community medicine so as to have the desired impact on population health.

The role of social policy as a primary determinant of population health was emphasized by Rudolf Virchow, a pathologist who is considered to be the father of social medicine.(1) McKeown also showed that infectious diseases had declined before the discovery and use of anti-microbial agents, as a result of better living standards and improved nutrition status which had increased people's capacity to combat infectious diseases.(2) However, specific biomedical approaches of preventive medicine gained pre-eminence with the advancement in the understanding of biological causes of diseases (microbes, nutrients, chemicals). Hence, in the later part of 19^th^ century, the practice of hygiene, that emphasized specific preventive measures such as personal cleanliness and environmental sanitation, became the main tool of public health for prevention and control of infectious diseases.

In the mid 20^th^ century, with the emergence of chronic non-communicable diseases, integration of the biomedical and sociological approaches formed the core philosophy of preventive and social medicine. The assimilation of family medicine with preventive and social medicine to meet the needs of primary heath care gave birth to community medicine in the health centers of South Africa.(3) The United Kingdom incorporated the management of health services as one of the functions of community medicine, rechristening community medicine as public health medicine.(4) According to Preston, this approach(5) enhanced access to primary medical care, which was made available to the masses by various disease prevention and control programs, and led to major declines in mortality in developing countries during the second half of the 20^th^ century.

Demographic transitions in Europe were triggered by the industrial revolution leading to socio-economic development whereas in the developing world the community medicine approach led to a decline in mortality and fertility. However, with advancing epidemiologic transition,(6) morbidity is rising despite a fall in the mortality. In the current situation, the traditional biomedical approach is not sufficient to stem the rise of chronic non-communicable diseases. Public health has evolved further to emphasize the role of social determinants, i.e., social and physical environments in shaping the lifestyle of populations. The Ottawa charter(7) on health promotion has also emphasized the primary role of social policy in health development. A multi-sectoral approach is required for making substantial gains in population health.

The academic growth of community medicine can take two directions, i.e., to develop into public health or family medicine. The focus of public health is to bring about changes at the policy level not only for prevention of disease but also for health promotion through organized actions at societal level, whereas family medicine has a thrust on delivery of preventive and curative primary health care service to families with their active participation. A family physician should be able to do more than what an MBBS doctor (general practitioner) can do. He/she should not only be able to deal with common medical, surgical, obstetrical, pediatric problems, and emergencies in a Primary or Community Health Center having facilities for conducting deliveries, admitting patients, and running round the clock emergency service but should also be able to manage health services at these levels. Five such multitasked physicians enabled to meet family health needs are a very cost effective substitute for five specialists at the Community Health Center.

As the role of health in socio-economic development is becoming clearer, governments are allocating more resources to public health. Hence, the requirement of public health human resources will increase manifold in the near future. Indian public health standards have specified the requirement of a public health professional in each of the community development blocks, the Integrated Disease Surveillance Project is recruiting an epidemiologist in every district of India, and the National Rural Health Mission has also created the position of public health manager in most health institutions. Therefore, institutional capacity for development of public health human resources by initiation of certificate, diploma and degree courses of one to two year duration for pre-service and in-service candidates from medical, nursing, laboratory, nutrition, environment, biology and social science streams should be accorded due priority. A three-year bachelor of public health course can be a good addition for augmenting the already depleted supervisory cadre in the public health system. Substantial restructuring of the curricula is required for development of competencies not only in epidemiology, health management, heath education, and health informatics, but also in public policy, health economics, environmental and occupational health, and health promotion. In the short-term, existing professional resources from allied disciplines can be co-opted to build the required capacity in community medicine and public health, two sides of the same coin.
